# Epidemiology of Hypoxic Community-Acquired Pneumonia in Children Under 5 Years of Age: An Observational Study in Northern India

**DOI:** 10.3389/fped.2021.790109

**Published:** 2022-02-09

**Authors:** Shally Awasthi, Tuhina Rastogi, Anuj Kumar Pandey, Chittaranjan Roy, Kripanath Mishra, Neelam Verma, Chandra Bhushan Kumar, Pankaj Kumar Jain, Rajesh Yadav, Abhishek Chauhan, Namita Mohindra, Ram Chandra Shukla, Monika Agarwal, Chandra Mani Pandey, Neera Kohli

**Affiliations:** ^1^Department of Pediatrics, King George's Medical University, Lucknow, India; ^2^Department of Community Medicine, Darbhanga Medical College and Hospital, Darbhanga, India; ^3^Department of Pediatrics, Darbhanga Medical College and Hospital, Darbhanga, India; ^4^Department of Pediatrics, Patna Medical College and Hospital, Patna, India; ^5^Department of Community Medicine, Uttar Pradesh University of Medical Sciences, Etawah, India; ^6^Department of Pediatrics, Uttar Pradesh University of Medical Sciences, Etawah, India; ^7^Department of Radio-Diagnosis, Dr. Ram Manohar Lohia Institute of Medical Sciences, Lucknow, India; ^8^Department of Radio-Diagnosis, Sanjay Gandhi Post Graduate Institute of Medical Sciences, Lucknow, India; ^9^Department of Radio-Diagnosis, Institute of Medical Sciences, Banaras Hindu University, Varanasi, India; ^10^Department of Community Medicine, King George's Medical University, Lucknow, India; ^11^Department of Biostatistics and Health Informatics, Sanjay Gandhi Postgraduate Institute of Medical Sciences, Lucknow, India; ^12^Department of Radio-Diagnosis, King George's Medical University, Lucknow, India

**Keywords:** hypoxic pneumonia, children, pallor, primary end-point pneumonia, India

## Abstract

**Background:**

Community-acquired pneumonia (CAP) is the leading cause of under-five mortality in India. An increased risk of mortality has been reported in cases of hypoxic pneumonia.

**Methods:**

The primary objective of this study was to assess the proportion of children aged 2–59 months, hospitalized with hypoxic CAP, as well as socio-demographic, clinical, and radiological features associated with it. The secondary objective was to determine the risk of mortality among hospitalized cases of hypoxic CAP. This prospective, observational study was conducted in four districts of Northern India, between January 2015 and April 2021. A hospital-based surveillance network was established. Inclusion criteria were as follows: (a) child between 2 and 59 months, (b) hospitalization with symptoms of WHO-defined CAP, (c) resident of project district, (d) illness of <14 days, and (e) child had neither been hospitalized for this illness nor recruited previously. Children whose chest x-rays (CXRs) were either unavailable/un-interpretable and those that received any dose of pneumococcal conjugate vaccine-13 were excluded. Hypoxic pneumonia was defined as oxygen saturation <90% on pulse oximetry or requiring oxygen supplementation during hospital stay.

**Results:**

During the study period, 71.9% (7,196/10,006) children of severe pneumonia were eligible for inclusion, of whom 35.9% (2,580/7,196) were having hypoxic pneumonia. Female gender and use of biomass fuel for cooking increased the odds of hypoxic CAP. Clinical factors like wheezing, pallor, tachypnea, low pulse volume, presence of comorbidity, general danger signs, severe malnutrition, and radiological finding of primary end-point pneumonia ± other infiltrates (PEP±OI) also increased the odds of hypoxic CAP in a conditional logistic regression model. Adjusted odds ratio for mortality with hypoxia was 2.36 (95% CI: 1.42–3.92).

**Conclusion:**

Almost one-third of cases hospitalized with severe CAP had hypoxia, which increased chances of mortality. Besides known danger signs, certain newer clinical signs such as pallor and wheezing as well as PEP+OI were associated with hypoxic CAP. Therefore, objective assessment of oxygen saturation must be done by pulse oximetry in all cases of CAP at the time of diagnosis.

## Introduction

Community-acquired pneumonia (CAP) is one of the leading causes of mortality among children under 5 years of age, globally as well as in India. In 2018, CAP accounted for the death of 0.8 million children globally (1), of whom 0.13 million were in India, killing more than 14 children every hour (1). Almost all of these deaths occur in cases of WHO-defined severe pneumonia (2). It has been estimated that 9–39% of children hospitalized for CAP in Asian countries have hypoxia (3). An increased risk of mortality has been reported in cases of hypoxic pneumonia (4, 5).

Presence of hypoxia can be measured non-invasively by pulse oximetry, which gives a value for oxygen saturation of hemoglobin. Value of oxygen saturation <90% has been taken as a cutoff for hypoxia and is an indicator for the need for administering supplemental oxygen (6). This oxygen saturation cutoff correlates well with clinical manifestations of respiratory distress (7–9). Hypoxia in CAP is largely due to diffusion–perfusion mismatch at the alveolar level (10). Lower respiratory tract infections, with bacteria such as *Streptococcus pneumoniae* (SP), *Haemophilus influenzae* type B (Hib), and *Staphylococcus aureus* as well as viruses, increase the chances of hypoxia (11, 12).

There are limited studies from India that report the prevalence of hypoxia in children with CAP and epidemiology associated with it (12, 13). Hence, as a primary objective, we analyzed data from our district-based, prospective, hospital surveillance of CAP; to assess the proportion of cases with hypoxic pneumonia and socio-demographic, clinical, and radiological features associated with it. The risk of mortality among these hospitalized cases of hypoxic pneumonia was determined as a secondary objective.

## Methods

Data were collected as a part of district-based, prospective, observational, hospital-based surveillance of CAP in children aged 2–59 months (14). This surveillance network was established in Lucknow and Etawah districts of Uttar Pradesh and Patna and Darbhanga districts of Bihar, Northern India. Included in this network were public and private hospitals in the district that admitted pediatric cases (14). Trained surveillance officers recruited cases from these network hospitals after parental consent (14). Inclusion criteria were as follows: (a) child between 2 and 59 months of age, (b) hospitalization with symptoms of WHO-defined CAP, (c) resident of project district, (d) illness of <14 days, and (e) child had neither been hospitalized for this illness nor recruited previously in our surveillance. Since 13-valent pneumococcal conjugate vaccine (PCV13) was introduced in 2017/2018 in a phased manner in India, in districts under our surveillance (15), children (2–59 months) who had received any dose of PCV13 were excluded from the analysis. Also excluded were cases with unavailable or un-interpretable chest x-rays (CXRs). From the hospital admission records, children in eligible age were screened for their suitability for inclusion. Details about the study have been published elsewhere (14, 16, 17).

The World Health Organization definition of CAP was used (18). Children with fast breathing with or without chest in-drawing were classified as “*pneumonia*”. Children were classified as “*severe pneumonia*” when they had cough or difficulty in breathing, plus at least one of the following: (a) central cyanosis or oxygen saturation <90% on pulse oximetry, (b) severe respiratory distress (e.g., grunting, very severe chest indrawing), (c) signs of pneumonia with a general danger sign: inability to breastfeed or drink, lethargy or unconscious, and convulsions. Tachypnea was defined by the WHO IMCI age-specific cutoffs of RR ≥50 breaths/min in children 2–11 months, and ≥40 breaths/min in children 12–59 months of age (19). Hypoxic pneumonia was defined as documented oxygen saturation <90% on pulse oximetry or requiring oxygen supplementation at any time during hospitalization (20). A child with weight-for-age (WAZ) score of ≤ -3 SD was considered to be having severe malnutrition and was calculated using WHO Anthro Survey Analyser (21).

After obtaining written, informed consent from the parents/legal guardians, trained surveillance officers abstracted demographic, anthropometric (weight and height), and clinical data from the hospital records. Clinical data were recorded by pre-existing trained hospital staff at the time of hospitalization. Socio-demographic information was obtained by face-to-face interviewing of parents/guardians. Surveillance officers noted clinical outcome (discharge or death) from the hospital logbook on follow-up. Detailed methodology of data collection has been published elsewhere (14, 16, 17). Immunization status of children (other than PCV) residing in our four study districts is given (Appendix Table A).

A hardcopy of CXR, if done on advice of the treating physician, was collected. CXRs were either analog or digital. All CXRs were digitalized and stored online. A panel of radiologists, trained using WHO methodology (22), interpreted CXRs by categorizing the quality of film as either “*interpretable*” or “*un-interpretable*”. If no comment was possible for radiological abnormality with respect to presence or absence of consolidation or pleural effusion or other infiltrates in a CXR, it was labeled as “*un-interpretable*” (23). Interpretable CXRs were categorized into (i) “Primary end-point pneumonia (PEP) only” or (ii) “other infiltrates (OI) only” or (iii) “both PEP and OI” (23). Standardized WHO case definition of PEP and OI was used (23) and has been reported elsewhere (17).

Data of more than 6 years (January 2015–April 2021) were analyzed to compare the socio-demographic, clinical, and radiological findings of hypoxic with non-hypoxic pneumonia. Children from all study districts were included in the primary analysis. To fulfill the secondary objective, association of mortality with hypoxic pneumonia among children (2–59 months) was evaluated.

### Statistical Analysis

We analyzed data of children aged 2–59 months hospitalized with CAP who fulfilled all inclusion criteria and had no exclusion criteria. Cases was categorized into two groups: “*hypoxic pneumonia*” and “*non-hypoxic pneumonia*”. We compared categorical as well as continuous variables among children with and without hypoxic pneumonia. Number and percentage were calculated for all categorical data. Mean and standard deviation (mean, SD) for normal continuous data and median and interquartile range (IQR) for non-normal continuous data were calculated. All statistical analysis was performed using statistical software (SPSS version 24). Chi-square test was used for comparison of categorical and Student's *t*-test for normal continuous data. Mann–Whitney *U* test was used for comparison of non-normally distributed data. A *p*-value of < 0.05 was taken as statistically significant using a two-tailed distribution. Likewise, the variables associated with mortality were also compared. Crude odds ratio (OR) with 95% confidence interval (CI) was calculated for association of mortality during hospitalization with hypoxic pneumonia.

Two conditional logistic regression (CLR) models were developed. The first model was to assess the association of hypoxic CAP with those independent variables that had univariate association with it with a *p*-value ≤ 0.1 and were clinically meaningful. Adjusted OR with 95% CI are being reported. In the second model, we assessed the association of mortality with hypoxia, adjusting for those variables that had univariate association with mortality with a *p*-value ≤ 0.1.

### Ethics

All sites obtained ethical clearance of the research project from their respective institutional ethical committee: (i) King George‘s Medical University, Lucknow vide letter no. 2800 Ethics/R Cell-14 dated November 22, 2014; (ii) U.P. Rural Institute of Medical Sciences & Research, Etawah vide letter no. 14/dean/RIMS & R/2015–16 dated April 25, 2015; (iii) Darbhanga Medical College & Hospital, Darbhanga vide letter no. 05/IEC/DMC dated February 19, 2015; and (iv) Patna Medical College and Hospital, Patna vide letter no. nil dated October 15, 2015. Parents/legal guardians of children provided written, informed consent for participation. Standardized procedures were followed for case enrolment and data collection. The study protocol and data collection forms are publicly available (16, 17).

## Results

**Figure 1:**
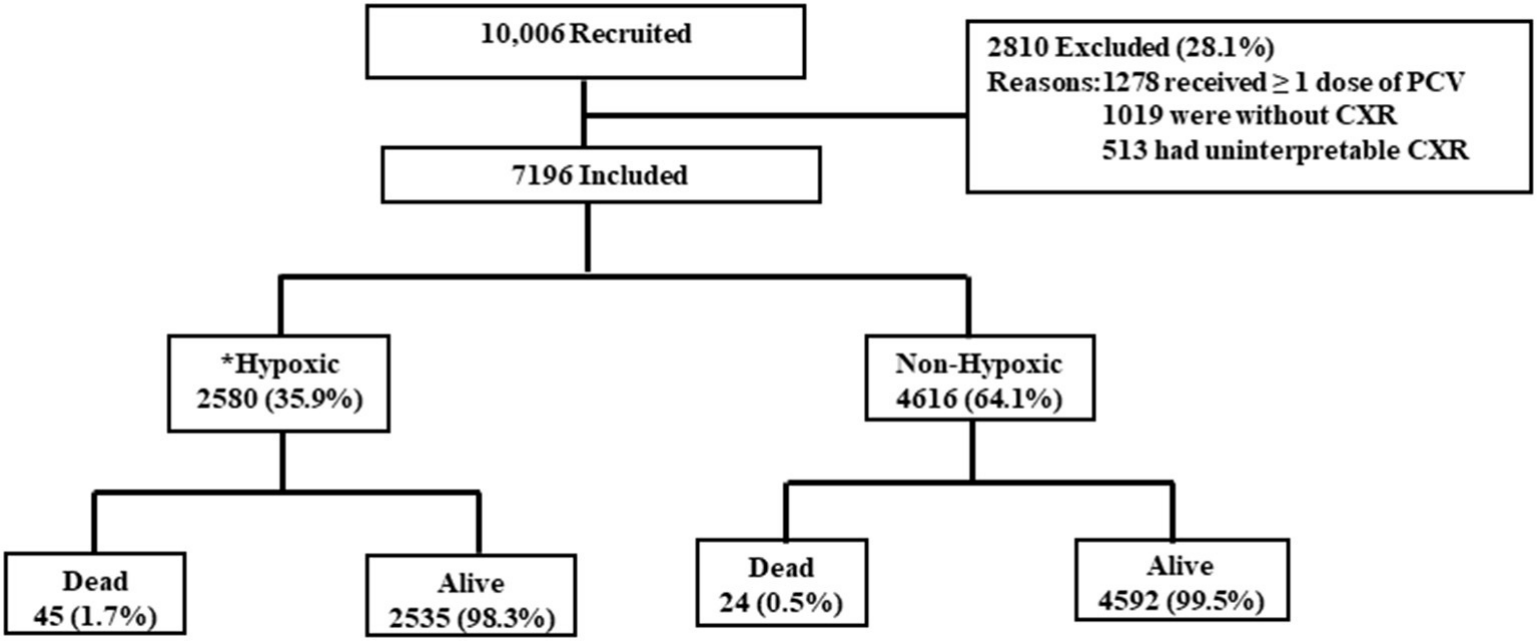
Children (2–59 months) hospitalized with community-acquired pneumonia from 2015 to 2021.

From January 2015 to April 2021, 10,006 children were recruited from the four districts. Of the recruited children, 28.1% (2,810/10,006) were excluded from the current analysis. The reasons for exclusion are given in Figure 1. Thereafter, the current analysis was done on 7,196 children of severe CAP. Among those, 35.9% (95% CI: 35.0–37.0%) (2,580/7,196) had hypoxic pneumonia. Among 2,580 children with hypoxic pneumonia, 85.5% (2,206/2,580) were diagnosed by pulse oximetry reading of <90% and the remaining 14.5% (374/2,580) by their need for supplemental oxygen during hospital stay. Site-wise distribution of children with hypoxic pneumonia was as follows: 28.8% (744/2,580) children in Lucknow, 19.3% (499/2,580) in Etawah, 26.1% (674/2,580) in Patna, and 25.7% (663/2,580) in Darbhanga district. Crude OR of death in cases of hypoxic pneumonia was 3.40 (95% CI, 2.07–5.59, *p* < 0.001).

Table 1 reports the univariate association of socio-demographic and clinical variables with hypoxic pneumonia. The variables that were significantly associated with hypoxic pneumonia were children aged 2-11 months, residing in rural areas, whose mothers had not received formal education (those that had never been enrolled in a government-recognized school), and households that used biomass fuel for cooking. Pallor and wheezing were proportionally increased in cases of hypoxic pneumonia than in controls. Mean respiratory rate (RR) of children (2–11 months) with hypoxic pneumonia was 60 breaths per minute (bpm), and that among children of age 12–59 months was 54 bpm. In the CLR model, RR was converted into a categorical variable as tachypnea (yes/no). Low pulse volume was significantly associated with hypoxic pneumonia. Among the clinical features, fast breathing and difficult breathing had significantly higher proportion in hypoxic pneumonia. All general danger signs had significantly higher proportion in hypoxic pneumonia (Table 1). In radiological findings, those with PEP±OI had significantly higher proportion in hypoxic pneumonia.

**Table 1:** Univariate association of clinical variables in cases with hypoxic community-acquired pneumonia.

**Characteristics of children** **column, ***N*** (%)**	**Total** ***N*** **= 7,196**	**Hypoxic** ***N*** **= 2,580**	**Non-hypoxic** ***N*** **= 4,616**	***p*** **-value**
Age (months) *n*, median (IQR)	7,196, 7.0 (4.0, 16.0)	2,580, 7.0 (3.0, 15.0)	4,616, 8.0 (4.0, 16.0)	<0.001
Weight-for-age *n*, mean (SD)	7,196, −1.5 (1.6)	2,580, −1.6 (1.6)	4,616, −1.4 (1.6)	<0.001
Height-for-age *n*, mean (SD)	7,196, −2.1 (1.9)	2,580, −1.9 (1.9)	4,616, −2.2 (1.9)	<0.001
Age (2–11 months), *n* (%)	4,736 (65.8)	1,764 (68.4)	2,972 (64.4)	0.001
Gender (female), *n* (%)	2,202 (30.6)	821 (31.8)	1,381 (29.9)	0.09
Residence (rural), *n* (%)	3,791 (52.7)	1,525 (59.1)	2,266 (49.1)	<0.001
Mother's education (without formal education), *n*/*N* (%)	2,522/7,153 (35.3)	988/2,566 (38.5)	1,534/4,587 (33.4)	<0.001
Biomass fuel for cooking, *n* (%)	3,244 (45.1)	1,291 (50.0)	1,953 (42.3)	<0.001
Pallor, *n* (%)	3,795 (52.7)	1,451 (56.2)	2,344 (50.8)	<0.001
Wheezing, *n*/*N* (%)	4,798/7,111 (67.5)	1,846/2,561 (72.1)	2,952/4,550 (64.9)	<0.001
Pulse volume (Low), *n*/*N* (%)	412/7,061 (5.8)	208/2,541 (8.2)	204/4,520 (4.5)	<0.001
*Comorbidities, *n* (%)	255 (3.5)	124 (4.8)	131 (2.8)	<0.001
**Respiratory rate by age**				
2–11 months *n*, mean (SD)	4,727, 56.5 (11.2)	1,763, 60.0 (10.8)	3,845, 54.5 (10.9)	<0.001
12–59 months *n*, mean (SD)	2,450, 50.2 (12.4)	813, 54.2 (12.0)	1,637, 48.2 (12.1)	<0.001
**Clinical features**				
Cough, *n* (%)	7,135 (99.2)	2,554 (99.0)	4,581 (99.2)	0.27
Fever, *n* (%)	6,452 (89.7)	2,313 (89.7)	4,139 (89.7)	0.98
Fast breathing, *n* (%)	6,404 (89.0)	2,431 (94.2)	3,973 (86.1)	<0.001
Difficult breathing, *n* (%)	6,981 (97.0)	2,528 (98.0)	4,453 (96.5)	<0.001
**General danger sign**				
Vomiting everything, *n* (%)	2,299 (31.9)	871 (33.8)	1,428 (30.9)	0.01
Lethargy, *n* (%)	3,246 (45.1)	1,295 (50.2)	1,951 (42.3)	<0.001
Convulsions, *n* (%)	353 (4.9)	217 (8.4)	136 (2.9)	<0.001
Inability to drink, *n* (%)	2,532 (35.2)	1,070 (41.5)	1,462 (31.7)	<0.001
Grunting, *n* (%)	5,658 (78.6)	2,154 (83.5)	3,504 (75.9)	<0.001
Severe malnutrition *n* (%)	1,123 (15.6)	463 (17.9)	660 (14.3)	<0.001
**Chest x-ray**, ***n*** **(%)**				
Normal/Other infiltrates	5,567 (77.4)	1,931 (74.8)	3,636 (78.8)	<0.001
Primary endpoint pneumonia ± Other infiltrates	1,629 (22.6)	649 (25.2)	980 (21.2)	

* *Comorbidities: Included congenital heart disease and history of fast breathing and cough ≥ 3 times in 6 months*.

Table 2 gives the results of the CLR to assess the association of hypoxic pneumonia with categorical variables. Gender (female), biomass fuel for cooking, symptoms of pallor and wheezing, tachypnea, low pulse volume, comorbidities, any danger sign of pneumonia, severe malnutrition, and those with PEP±OI in CXRs had increased adjusted OR for hypoxic pneumonia. Comorbidities present were congenital heart disease (1.4%, 102/7,196) and history of fast breathing and cough ≥ 3 times in 6 months (2.2%, 159/7,196).

**Table 2:** Conditional logistics regression to assess association of hypoxic community pneumonia with sociodemographic, clinical, and radiological variables.

**Variables**	**Hypoxic/Non-hypoxic^**ref**^**	***p*** **-value**
	**Adjusted odds ratio** **(95% confidence interval)**	
Age (2–11 months)	1.31 (1.18–1.47)	<0.001
Gender (female)	1.14 (1.02–1.27)	0.02
Biomass fuel for cooking	1.19 (1.07–1.32)	0.001
Wheezing	1.40 (1.25–1.56)	<0.001
Pallor	1.22 (1.11–1.35)	<0.001
Tachypnea	2.40 (2.10–2.74)	<0.001
Pulse volume (low)	1.92 (1.56–2.37)	<0.001
*Comorbidities	1.53 (1.18–1.99)	0.002
Any danger sign of pneumonia	1.62 (1.26–2.08)	<0.001
^β^Severe malnutrition	1.29 (1.12–1.49)	<0.001
**Chest x-ray**		
Normal/Other Infiltrates^ref^		
Primary endpoint pneumonia ± Other infiltrates	1.17 (1.04–1.32)	0.01

* *Comorbidities: Included congenital heart disease and history of fast breathing and cough ≥ 3 times in 6 months*.

β *Severe malnutrition: weight-for-age (WAZ) score of ≤ -3 SD*.

Table 3 reports the sociodemographic, clinical associates of mortality due to CAP. No formal education in mothers, biomass fuel for cooking, comorbidities, tachypnea, low pulse volume, hypoxic pneumonia, severe malnutrition, and PEP ± OI were significantly associated with mortality.

**Table 3:** Comparison of sociodemographic and clinical variables of cases with and without adverse outcome (death).

**Characteristics of children**	**Dead**	**Alive**	***p*** **-value**
**Column, *n* (%)**	***N*** **= 69**	***N*** **= 7,127**	
Age (2–11 months), *n* (%)	51 (73.9)	4,685 (65.7)	0.15
Gender (female), *n* (%)	28 (40.6)	2,174 (30.5)	0.07
Residence (rural), *n* (%)	42 (60.9)	3,749 (52.6)	0.17
Mother's education (without formal education), *n*/*N* (%)	35/69 (50.7)	2,487/7,084 (35.1)	0.007
Biomass fuel for cooking, *n* (%)	44 (63.8)	3,200 (44.9)	0.002
Pallor, *n* (%)	29 (42.0)	3,766 (52.8)	0.07
Wheezing, *n*/*N* (%)	40/66 (60.6)	4,758/ (67.5)	0.23
*Comorbidities, *n* (%)	11 (15.9)	244 (3.4)	<0.001
Pulse volume (low), *n*/*N* (%)	9/69 (13.0)	403/6,992 (5.8)	0.01
Tachypnea, *n* (%)	66 (95.7)	5,397 (75.7)	<0.001
Any danger sign of pneumonia, *n* (%)	67 (97.1)	6,726 (94.4)	0.33
Hypoxic pneumonia, *n* (%)	45 (65.2)	2,535 (35.6)	<0.001
Severe malnutrition, *n* (%)	35 (50.7)	1,088 (15.3)	<0.001
**Chest x-ray**, ***n*** **(%)**		
Normal/Other infiltrates	32 (46.4)	5,535 (77.7)	<0.001
Primary endpoint pneumonia ± Other infiltrates	37 (53.6)	1,592 (22.3)	

* *Comorbidities: Congenital heart disease and history of fast breathing and cough ≥ 3 times in 6 months*.

Table 4 gives the result of the CLR of association of hypoxic pneumonia with mortality, controlling for sociodemographic, clinical, and radiological variables. Children with hypoxic pneumonia were at increased odds of hospital mortality (adjusted OR 2.36, 95% CI: 1.42–3.92).

**Table 4:** Conditional logistics regression to assess association of mortality with hypoxic CAP controlling for sociodemographic, clinical, and radiological variables.

**Variables**	**Dead/alive^**ref**^**	***p*** **-value**
	**Adjusted odds ratio** **(95% confidence interval)**	
Age (2–11 months)	1.21 (0.69–2.13)	0.50
Gender (female)	1.58 (0.96–2.60)	0.07
Biomass fuel for cooking	1.85 (1.11–3.08)	0.02
Tachypnea	4.80 (1.49–15.45)	0.009
*Comorbidities	3.45 (1.74–6.86)	<0.001
Pulse volume (low)	1.94 (0.93–4.07)	0.08
Severe malnutrition	4.65 (2.71–7.97)	<0.001
Hypoxic pneumonia	2.36 (1.42–3.92)	0.001
**Chest x-ray**		
Normal/other infiltrates^ref^		
Primary endpoint pneumonia ± Other infiltrates	3.07 (1.87–5.02)	<0.001

* *Comorbidities: Congenital heart disease and history of fast breathing and cough ≥ 3 times in 6 months*.

## Discussion

From the data of a prospective, hospital surveillance network set up in four districts of Northern India (14), data analysis was done among cases of CAP, which were hospitalized over a period of 6 years (January 2015–April 2021). We found the prevalence of hypoxic pneumonia to be 35.9% (2,580/7,196). Increased odds of hypoxic pneumonia were found among infants (2–11 months), females, with use of biomass fuel for cooking, and presence of pallor, wheezing, tachypnea, low pulse volume, severe malnutrition, and any general danger sign of illness (inability to breastfeed or drink, lethargy or unconscious, and convulsions), and those with PEP ± OI on CXRs. Children with hypoxic pneumonia were at increased odds of hospital mortality.

Hypoxia was defined as oxygen saturation <90% or use of supplemental oxygen for the treatment of CAP. Studies conducted in Nepal (24), Nigeria (25), and Sudan (26) have also used oxygen saturation <90% to define hypoxia at sea level, and this is similar to our study. The extent of compromise of lung, as a result of pneumonia, is due to altered alveolar ventilation diffusion and resultant intra-pulmonary shunting (27). It manifests as reduced oxygen saturation and clinical signs of respiratory distress or failure. These are treated with supplemental oxygen. Hence, our definition of hypoxia in the current study is robust and has been used by others too (20).

Our study found that 35.9% (2,580/7,196) of hospitalized cases had hypoxic pneumonia. The Pneumonia Etiology Research for Child Health (PERCH) study, conducted in 7 countries to assess the etiology of and risk factors for severe and very severe pneumonia in children (1–59 months), reported the overall prevalence of hypoxia to be 35.8% (1,423/3,981) and 42.3% (748/1,769) among children with abnormal CXRs (28). Another observational study from India (12) conducted in a similar setting found hypoxia in 40% (54/135) of the patients, and of them, 37% (20/54) had pneumonia with lower chest indrawing and 63% (34/54) had severe pneumonia (12). Studies conducted in Nepal (24), Nigeria (25), and Sudan (26) have reported prevalence of hypoxemia to be 38.7, 41.5, and 42.7%, respectively, which is similar to that found by us. However, a study from Papua New Guinea reported much higher prevalence of hypoxemia (70%) (29).

In our study, cases with hypoxic pneumonia had significantly higher odds for hospital mortality and the odds of death were 2.36 times higher among children with hypoxic pneumonia than those without. Our results are similar to the studies from Kenya (30), Zambia (31), and Gambia (32) that reported that the odds of death to be 4.3 to 5.6 times higher in children with hypoxia than in those without. A systematic review and meta-analysis on 12 observational studies based on 13,936 under-five children in lower- and middle-income countries reported that the presence of hypoxia (oxygen saturation <90%) caused a fivefold increase in the odds of death from acute lower respiratory infections (33).

We found that presence of PEP±OI in CXRs was positively associated with presence of hypoxia as well as with odds of mortality. The PERCH study reported that cases with abnormal CXRs were more likely than those with normal CXRs to have hypoxemia (45 vs. 26%) (34). Furthermore, a cohort study conducted in a pediatric emergency department in India found that oxygen saturation <92% was the strongest predictor of radiographically confirmed pneumonia (35). Radiological pneumonia has been associated with infection with SP (9). This is a possibility in our study also as immunization against SP started in 2017 only while immunization against *Hib* was there in Expanded Program of Immunization of the Government of India since 2016 (36).

Hypoxic pneumonia can be detected using clinical signs, pulse oximetry, or blood gas analysis (37). A systematic review conducted in 2020 to update the management of CAP found that certain clinical features of respiratory failure/distress (nasal flaring, grunting, and head nodding, lower chest indrawing, and central cyanosis) as well as some general danger signs can be used as indicators for hospitalization and predictors of hypoxia as well as mortality due to it (38).

Literature from Asia (20) and Africa (39, 40) indicates that PCV13 is effective against hypoxic pneumonia. A prospective study conducted in a single tertiary-care hospital in Lao People's Democratic Republic found a 37% reduction in hypoxic pneumonia post PCV-13 introduction while studies from Malawi and Gambia reported a 47 and 61% reduction, respectively, in hypoxic pneumonia after PCV13 introduction (39, 40). Since SP is the cause of CAP in areas where there is good coverage with Hib (9), expedited coverage of PCV13 may also help in reduction of hypoxic pneumonia and consequently childhood mortality.

In our study, additional clinical features of pallor and wheezing were significantly associated with presence of hypoxic pneumonia. Pallor was also found to be significantly associated with hypoxic pneumonia in a study conducted in India (12) and also in a Nigerian study (41). Therefore, pallor must be included as an additional general danger sign of pneumonia. Further research in this direction is needed.

We found that the presence of CXR abnormalities is associated with increased odds of hypoxia as well as mortality. Hence CXR should be recommended in cases of severe CAP so that those with abnormalities can be triaged for referral to higher health facilities. This is also in accordance with Pediatric Infectious Diseases Society and the Infectious Diseases Society of America guidelines for the management of CAP in infants and children, where CXR is recommended in cases of hypoxemia (suspected or documented) or significant respiratory distress (37).

In our study, 35.9% (2,580/7,196) children had hypoxic pneumonia. We also found that the presence of hypoxic CAP increased the odds of hospital mortality. One of the reasons for high proportion of children with CAP identified as having hypoxia may be delayed recognition of danger signs by the caregivers and by the healthcare workers. Literature from India (42–44) have reported how delayed recognition of danger signs (42, 44) and delayed healthcare-seeking (42, 43) by the caregivers increased the severity of CAP and caused high mortality. Therefore, early recognition of CAP by the caregivers and healthcare workers must be encouraged.

### Strengths and Limitations

This study was a district-based surveillance of hospitalized cases of pediatric CAP. Standardized definitions were used for recruitment (2). Use of data from four districts located in two states of Northern India ensured generalizability of our findings. Our data were collected from a large network of public and private hospitals over a period of more than 6 years (January 2015–April 2021). It increased reliability of the data. CXRs were interpreted using a standardized WHO-recommended methodology (21), by a panel of trained radiologists (17), and this ensured both internal and external validity.

In our study, pulse oximetry readings were abstracted from the hospital records by the surveillance officers. Pulse oximeters used for measuring oxygen saturation were not provided by the project since it was a pragmatic observational study. In our study, hypoxia was defined as oxygen saturation <90% or use of supplemental oxygen for the treatment of CAP. While the pulse oximetry readings are objective, the use supplemental oxygen for treatment of CAP by doctors was largely based on subjective assessment and could have resulted in mis-classification bias. During analysis, we excluded a small proportion (16.7%) of children without CXRs.

## Conclusion

Almost one-third of cases hospitalized with severe CAP had hypoxia, which increased chances of mortality. Besides the known danger signs, certain newer clinical signs such as pallor and wheezing as well as PEP ± OI on CXR are associated with hypoxic CAP. Therefore, objective assessment of oxygen saturation must be done by pulse oximetry in all cases of CAP at the time of diagnosis. Modification of standard care guidelines of severe CAP can be considered with introduction of routine chest radiography.

## Data Availability Statement

The original contributions presented in the study are included in the article, further inquiries can be directed to the corresponding author/s.

## Ethics Statement

The studies involving human participants were reviewed and approved by the Ethics Review Committee of three sites, details of which are as follows: (i) King George‘s Medical University, Lucknow vide letter no. 2800 Ethics/R Cell-14 dated 22nd November 2014. (ii) Darbhanga Medical College and Hospital, Darbhanga vide letter no. 05/IEC/DMC dated 19th February 2015. (iii) Patna Medical College and Hospital, Patna vide letter no. nil dated 15th October 2015. (IV) U.P. Rural Institute of Medical Sciences and Research, Etawah vide letter no. 14/dean/RIMS and R/2015-16 dated 25th April, 2015. The caregivers/guardians of children signed the written, informed consent for participation in the study.

## Author Contributions

SA conceived and designed the study. SA, TR, MA, CR, KM, NV, CK, PJ, and RY supervised data acquisition. Interpretation of chest x-rays was done by AC, NM, RS, and NK. CP and AP conducted the statistical analysis of the data and data management. The paper was written by SA, TR, CP, and AP. All authors were involved in revising the work and approved the final submission.

## Funding

This study was supported by the Bill & Melinda Gates Foundation (https://www.gatesfoundation.org/) *via* Grant No. OPP1189869/INV-006521 KGMU.

## Conflict of Interest

The authors declare that the research was conducted in the absence of any commercial or financial relationships that could be construed as a potential conflict of interest.

## Publisher's Note

All claims expressed in this article are solely those of the authors and do not necessarily represent those of their affiliated organizations, or those of the publisher, the editors and the reviewers. Any product that may be evaluated in this article, or claim that may be made by its manufacturer, is not guaranteed or endorsed by the publisher.
