# Supplementary material for: Epidemiology of Hypoxic Community-Acquired Pneumonia in Children Under 5 Years of Age: An Observational Study in Northern India
Source: Front Pediatr. 2022 Feb 9;9:790109. doi: 10.3389/fped.2021.790109 (PMC8863665; doi:10.3389/fped.2021.790109)
Supplement: Supplementary file 1 [file Table_1.docx]

**Web-appendix Table A: Immunization Status (other than PCV) in four study districts**

| Immunization Indicators | Lucknow District^1^ | Etawah District^2^ | Darbhanga District^3^ | Patna District ^4^ |
| --- | --- | --- | --- | --- |
| Children (12-23 months) fully immunized (BCG, measles, and 3 doses each of polio and DPT) (%) | 58.8 | 53.8 | 52.9 | 69.7 |
| Children (12-23 months) months who have received BCG (%) | 92.8 | 86.1 | 91.5 | 88.8 |
| Children (12-23 months) who have received 3 doses of polio vaccine (%) | 64.6 | 67.6 | 65.3 | 74.8 |
| Children (12-23 months) who have received 3 doses of DPT vaccine (%) | 72.2 | 62.8 | 72.0 | 85.1 |
| Children (12-23 months) who have received measles vaccine (%) | 79.9 | 66.9 | 75.4 | 84.2 |
| Children (12-23 months) who have received 3 doses of Hepatitis B vaccine (%) | 61.1 | 56.8 | 59.8 | 76.3 |

References:

1. National Family Health Survey – 4: District Fact Sheet-Lucknow: Available at: <http://rchiips.org/nfhs/FCTS/UP/UP_Factsheet_157_Lucknow.pdf>. Accessed on 2^nd^ Dec 2021
2. National Family Health Survey – 4: District Fact Sheet-Etawah: Available at: <http://rchiips.org/nfhs/FCTS/UP/UP_Factsheet_161_Etawah.pdf>. Accessed on 2^nd^ Dec 2021
3. National Family Health Survey – 4: District Fact Sheet Darbhanga Available at: <http://rchiips.org/nfhs/FCTS/BR/BR_FactSheet_215_Darbhanga.pdf>. Accessed on 2^nd^ Dec 2021
4. National Family Health Survey – 4: District Fact Sheet Patna Available at: <http://rchiips.org/nfhs/FCTS/BR/BR_FactSheet_230_Patna.pdf>. Accessed on 2^nd^ Dec 2021
